# Clinical Experience With Preventing Chemotherapy-Induced Neutropenia in Breast Cancer Patients With Different Timings of Pegylated Granulocyte Colony-Stimulating Factor (PEG-G-CSF) Injection: A Case Series

**DOI:** 10.7759/cureus.91258

**Published:** 2025-08-29

**Authors:** Yohei Tominaga, Koji Furukawa

**Affiliations:** 1 Department of Thoracic and Breast Surgery, Faculty of Medicine, University of Miyazaki, Miyazaki, JPN; 2 Department of Cardiovascular Surgery, Faculty of Medicine, University of Miyazaki, Miyazaki, JPN

**Keywords:** breast cancer chemotherapy, fibrile neutropenia, granulocyte colony-stimulating factor (g-csf), heterochronic follow-up, optimal timing

## Abstract

Pegfilgrastim (pegylated granulocyte colony-stimulating factor, PEG-G-CSF) is commonly used as prophylaxis for febrile neutropenia (FN) in high-risk chemotherapy regimens for breast cancer. However, the optimal timing of PEG-G-CSF injections has not been established, despite several investigations into the subject. In this study, patients received epirubicin-based breast cancer chemotherapy and underwent routine blood tests on days seven or eight of initial chemotherapy to assess the risk of FN. Four patients experienced significant decreases in white blood cell (WBC) and neutrophil (NE) counts.

To maintain patient safety and relative dose intensity (RDI), the dose was reduced in the second cycle, and PEG-G-CSF administration was moved from day three to day four, as administering PEG-G-CSF within 24 hours to prevent FN is thought to be ineffective. This theory is based on the fact that the WBC and NE expanded by PEG-G-CSF were killed by the remaining chemotherapy. Therefore, we hypothesized that in the next second cycle, administering PEG-G-CSF one day later (day four) after chemotherapy might be effective for these patients. Furthermore, for patients whose blood tests on days seven or eight of the second cycle showed an increase in WBC and NE counts, the chemotherapy dose was increased in the third cycle.

Patients with breast cancer (age range: 41-71 years) were assigned to receive PEG-G-CSF on day three or four of a three-week epirubicin and cyclophosphamide-based chemotherapy regimen (dose-dense epirubicin and cyclophosphamide; 5-fluorouracil, epirubicin, and cyclophosphamide; or basic epirubicin and cyclophosphamide) using heterochronic timing. We then compared WBC and NE counts in the same individuals over time. In all four cases, WBC and NE counts on day seven or eight were much greater with PEG-G-CSF injection on day four than with injection on day three. As per heterochronic follow-up, which has not been reported previously, day four injection of PEG-G-CSF appears more effective than day three injection for preventing neutropenia. However, due to the small number of cases in this series and the confounding factor of the chemotherapy dose in the third cycle being approximately 8% less than that in the first cycle in Case 2, it is difficult to generalize our findings. Hence, future studies with a longitudinal follow-up involving a larger number of cases are required.

## Introduction

Pegfilgrastim (pegylated granulocyte colony-stimulating factor, PEG-G-CSF) is routinely used as prophylaxis for febrile neutropenia (FN) during chemotherapy regimens for breast cancer at high risk of causing FN. However, even though PEG-G-CSF is highly effective in preventing FN, 2.8-16% of cases develop FN after chemotherapy [1,2]. FN is a serious adverse event in myelosuppressive chemotherapy that usually results in hospitalization and a need for intravenous antibiotics and is potentially life-threatening. FN may result in reductions to the doses of anticancer drugs, delays in chemotherapy, or even discontinuation of chemotherapy. Employing optional timing for PEG-G-CSF injection could reduce the risk of FN. Administration at 24-72 hours (or 24-96 hours) after chemotherapy injection is generally recommended to prevent FN [3,4], but the optimal timing of PEG-G-CSF injection has not been established, even though several reports on the timing of G-CSF injection have been published. Hence, this series, involving patients who received epirubicin-based breast cancer chemotherapy and underwent routine blood tests on days seven or eight of initial chemotherapy to assess the risk of FN, aims to address that gap in the literature.

## Case presentation

Patients and methods

Patients in this series were four women, ranging in age from 41 to 72 years. Pathological diagnosis was invasive ductal carcinoma in three cases (one case each of stage I, stage IIA, and stage IIB) and invasive lobular carcinoma in the remaining (stage IIA). Chemotherapy regimens comprised FEC100 (5-fluorouracil [5FU] 500 mg/m^2^, epirubicin 100 mg/m^2^, and cyclophosphamide 500 mg/m^2^, every three weeks) in one patient, ddEC (dose-dense epirubicin 90 mg/m^2^ and cyclophosphamide 600 mg/m^2^, every two weeks) in two patients and basic EC therapy (epirubicin 90 mg/m^2^ and cyclophosphamide 600 mg/m^2^, every three weeks) in one patient. The chemotherapy was used as adjuvant chemotherapy in three cases (Cases 1, 3, and 4) and as neoadjuvant chemotherapy in the remaining case (Case 2). 

PEG-G-CSF was injected on day three in the first cycle or day 4 in the subsequent cycles to prevent FN in each case. To evaluate the condition of the patient and the risk of FN, we measured WBC and NE counts before chemotherapy (day zero or one) and on day seven or eight after chemotherapy [5]. If the NE count was <500/mm^3^ on days seven or eight, additional blood tests were performed to assess WBC and NE counts.

Design of Chemotherapy and PEG-G-CSF Treatment

For the first cycle of these regimens, PEG-G-CSF was injected on day three after chemotherapy in the hospital. If these cell counts were low (e.g., WBC < 1000/mm^3^ or NE <500/mm^3^) on day seven or day eight, the dose of chemotherapy was reduced by approximately 10 to 20% and the timing of PEG-G-CSF injection was changed to day four after two cycles of the same chemotherapy regimen. If WBC and NE counts were increased on day seven or day eight in the second cycle of chemotherapy, we resumed the original dose or increased the dose of chemotherapy for the third and fourth cycles.

Results

Case 1: A 55-Year-Old Female

The patient with left-sided breast cancer had undergone a partial mastectomy and a sentinel lymph node biopsy. Pathological analysis showed invasive ductal carcinoma with ductal spread, resulting in pT1cN1mi (1/3) M0 stage IIA. Immunohistochemistry showed the following findings: estrogen receptor (ER)+ (Allred score: total score [TS] 6); progesterone receptor (PgR)+ (TS 5); human epidermal growth factor receptor 2 (HER2)+ (3+); and Ki67 labeling index (35%). Height, weight, and body surface area before starting chemotherapy were as follows: 149 cm, 47 kg, and 1.39 m^2^ (according to the Du Bois's formula), respectively. ECOG (Eastern Cooperative Oncology Group) performance status was 0.

Laboratory data before breast surgery and chemotherapy are provided in Table 1. These data indicated that she was a relatively healthy patient, and preoperative chemotherapy was therefore not considered likely to be problematic.

**Table 1:** Laboratory data on admission - Case 1 ALP: alkaline phosphatase; ALT: alanine aminotransferase; APTT: activated partial thromboplastin time; AST: aspartate aminotransferase; CEA: carcinoembryonic antigen; CRP: C-reactive protein; PT: prothrombin time; PT-INR: prothrombin time-international normalized ratio; RBC: red blood cells; WBC: white blood cells; CA 15-3: cancer antigen 15-3

Lab data		
Complete blood count	Value	Reference range
WBC	4930	3300-8600/mm^3^
RBC	3.54 x 10^6^	3.86-4.92 x 10^6^/mm^3^
Hemoglobin	12.7	11.6-14.8 g/dL
Platelets	20.2 x 10^4^	15.8-34.8 x 10^4^/mm^3^
Differential WBC (count)	Value	Reference range
Neutrophils	2830	NA
Lymphocytes	1840	NA
Monocytes	210	NA
Eosinophils	40	NA
Basophils	10	NA
Differential WBC (%)	Value	Reference range
Neutrophils	57.4	40-72 (%)
Lymphocytes	37.3	18-59 (%)
Monocytes	4.3	0.0-8.0 (%)
Eosinophils	0.8	0.0-6.0 (%)
Basophils	0.2	0.0-2.0 (%)
Coagulation	Value	Reference range
PT	10.3	10.5-13.5 sec
PT-INR	0.97	0.85-1.15
APTT	26.3	24.3-36.0 sec
Urinalysis	Value	Reference range
WBC	(-)	NA
Nitrite	(-)	NA
Urobilinogen	(+/-)	NA
Protein	(-)	NA
pH	5.5	5.0-7.5
Blood	(2+)	NA
Specific gravity	1.023	1.008-1.034
Ketones	(-)	NA
Bilirubin	(-)	NA
Glucose	(-)	NA
Blood chemistry	Value	Reference range
Total protein	7.2	6.6-8.1 g/dL
Albumin	4.4	4.1-5.1 g/dL
Total bilirubin	0.4	0.4-1.5 mg/dL
ALT	16	13-30 U/L
AST	14	7-23 U/L
ALP	148	106-322 U/L
Gamma-glutamyl transferase	22	9-32 U/L
Creatinine	0.57	0.46-0.79 mg/dL
Blood urea nitrogen	15	8–20 mg/dL
Uric acid	3.1	2.6-5.5 mgl/dL
Glucose	148 (95)	70-99 mg/dL
Sodium	141	136-145 mEq/L
Potassium	4	3.5-5.0 mEq/L
Chloride	106	95-105 mEq/L
Total calcium	9.4	8.8-10.1 mg/dL
Amylase	64	44-132 U/L
Creatine phosphokinase	52	41-153 U/L
CRP	0.09	0.00-0.14 mg/dL
Tumor marker	Value	Reference range
CEA	1.7	0.0-5.0 ng/mL
CA 15-3	5.9	0.0-31.3 U/mL

We initially used FEC100 therapy (5FU 650 mg/body [470 mg/m^2^, 94% dose], epirubicin 130 mg/body [94 mg/m^2^, 94% dose], cyclophosphamide 650 mg/body [470 mg/m^2^, 94% dose]) as adjuvant chemotherapy with PEG-G-CSF injection on day three. Concomitant medications included palonosetron hydrochloride 0.75 mg intravenously and dexamethasone 9.9 mg intravenously on day one. Oral aprepitant was used on day one at 125 mg, and on days two and three at 80 mg. Oral dexamethasone was used at 8 mg after breakfast and lunch on days two to four. These concomitant medications were used in all cycles.

**Figure 1:**
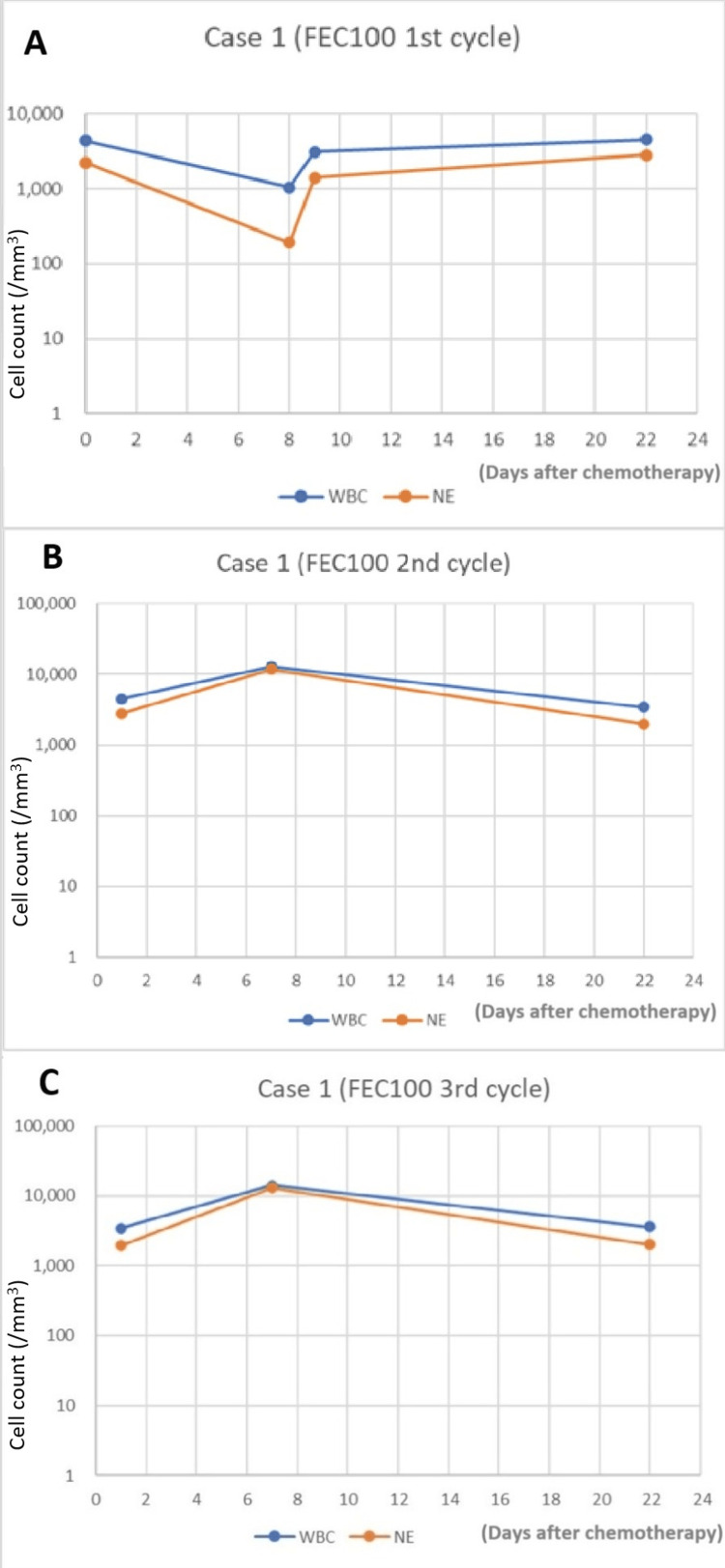
WBC and NE in Case 1 after PEG-G-CSF injection on day three (A) or day four (B and C) (A) FEC100, 1st cycle. (B) FEC100, 2nd cycle. (C) FEC100, 3rd cycle FEC100: 5-fluorouracil 500 mg/m^2^, epirubicin 100 mg/m^2^, and cyclophosphamide 500 mg/m^2; ^NE: neutrophil; PEG-G-CSF: pegylated granulocyte colony-stimulating factor; WBC: white blood cells

After the first round of FEC100 therapy, the patient reported mild fatigue and loss of appetite. These symptoms resolved with oral antiemetics. On day eight of the first cycle, blood testing was performed as a routine check for the first FEC100 therapy, revealing marked decreases in WBC and NE counts to 1,050/mm^3^ and 170/mm^3^, respectively (Figure 1A). To prevent FN, the antibiotic LVFX (levofloxacin) was administered for three days. The next day, both WBC and NE counts had increased without administration of the G-CSF reagent.

In the second cycle of FEC100 chemotherapy with an 8% reduction in dose (5FU 600 mg/body [430 mg/m^2^; 86% dose], epirubicin 120 mg/body [86 mg/m^2^; 86% dose], cyclopshosphamide 600 mg/body [430 mg/m^2^; 86% dose]) and PEG-G-CSF injection on day four, we observed tremendous increases in both WBC and NE counts, to 12,900/mm^3^ and 11,700/mm^3^, respectively, on day eight of the cycle (Figure 1B). Mild fatigue and anorexia appeared during the first several days of the second cycle, but again resolved promptly with oral antiemetics.

In the third cycle of FEC100 chemotherapy with the same dose as the first cycle (5FU 650 mg/body [470 mg/m^2^; 94% dose], epirubicin 130 mg/body [94 mg/m^2^; 94% dose], cyclopshosphamide 650 mg/body [470 mg/m^2^; 94% dose]) and PEG-G-CSF injection on day four, we observed high WBC and NE counts of 14,200/mm^3^ and 13,100/mm^3^, respectively, on day seven of the cycle (Figure 1C). The third cycle displayed a similar clinical course to the second. No fever was observed during any of the three cycles.

Monocyte counts measured on day eight of the first cycle were similar to those measured on day seven of the second and third cycles (Table 2). With regular outpatient visits and examinations, the patient has remained alive for five years since surgery without recurrence of the breast cancer.

**Table 2:** Comparisons of monocytes in Case 1 at different times of PEG-G-CSF (day three of cycle [c]1 of FEC therapy or day four of c2 and c3 of FEC therapy) FEC: fluorouracil, epirubicin, and cyclophosphamide; PEG-G-CSF: pegylated granulocyte colony-stimulating factor

Comparisons of monocytes						
Day after CT (c1)	0	1	7	8	9	22
Monocytes (%)	3.7	NA	NA	6.7	12.6	9.5
Monocytes (/mm^3^)	162	NA	NA	70	389	430
Day after CT (c2)	0	1	7	8	9	22
Monocytes (%)	NA	9.5	0.5	NA	NA	13
Monocytes (/mm^3^)	NA	430	65	NA	NA	450
Day after CT (c3)	0	1	7	8	9	22
Monocytes (%)	NA	13	0.3	NA	NA	10.8
Monocytes (/mm^3^)	NA	450	42	NA	NA	390

Case 2: A 41-Year-Old Female

The patient with left-sided breast cancer had undergone a reserved partial mastectomy and a sentinel lymph node biopsy. Pathological analysis suggested invasive ductal carcinoma, pT2N1M0 stage IIB. Immunohistochemistry showed the following findings: ER+ (Allred score: TS 8); PgR+ (TS 6); and HER2- (0). Height, weight, and body surface area before chemotherapy were as follows: 160 cm, 43.6 kg, and 1.42 m^2^ (Du Bois formula), respectively. ECOG performance status was 0.

Laboratory data before neoadjuvant chemotherapy are shown in Table 3. These data all suggested that the patient was relatively healthy and that preoperative chemotherapy was unlikely to present a problem.

**Table 3:** Laboratory data on admission - Case 2 ALP: alkaline phosphatase; ALT: alanine aminotransferase; APTT: activated partial thromboplastin time; AST: aspartate aminotransferase; CEA: carcinoembryonic antigen; CRP: C-reactive protein; PT: prothrombin time; PT-INR: prothrombin time-international normalized ratio; RBC: red blood cells; WBC: white blood cells; CA 15-3: cancer antigen 15-3

Lab data		
Complete blood count	Value	Reference range
WBC	7070	3300-8600 /mm^3^
RBC	4.36 x 10^6^	3.86-4.92 x 10^6^ /mm^3^
Hemoglobin	13.7	11.6-14.8 g/dL
Platelets	20.2 x 10^4^	15.8-34.8 x 10^4^ /mm^3^
Differential WBC (count)		
Neutrophils	4770	NA
Lymphocytes	2060	NA
Monocytes	160	NA
Eosinophils	20	NA
Basophils	20	NA
Differential WBC (%)	Value	Reference range
Neutrophils	67.5	40-72 (%)
Lymphocytes	29.1	18-59 (%)
Monocytes	2.8	0.0-8.0 (%)
Eosinophils	0.3	0.0-6.0 (%)
Basophils	0.3	0.0-2.0 (%)
Coagulation	Value	Reference range
PT	10.7	10.5-13.5 sec
PT-INR	1.01	0.85-1.15 sec
APTT	27.3	24.3-36.0 sec
Urinalysis	Value	Reference range
WBC	(-)	NA
Nitrite	(-)	NA
Urobilinogen	(+/-)	NA
Protein	(+/-)	NA
pH	6	5.0-7.5
Blood	(-)	NA
Specific gravity	1.022	1.008-1.034
Ketones	(-)	NA
Bilirubin	(-)	NA
Glucose	(-)	NA
Blood chemistry	Value	Reference range
Total protein	7.2	6.6-8.1 g/dL
Albumin	4.7	4.1-5.1 g/dL
Total bilirubin	0.8	0.4-1.5 mg/dL
ALT	20	13-30 U/L
AST	11	7-23 U/L
ALP	151	106-322 U/L
Gamma-glutamyl transferase	36	9-32 U/L
Creatinine	0.5	0.46-0.79 mg/dL
Blood urea nitrogen	12	8–20 mg/dL
Uric acid	3.8	2.6-5.5 mgl/dL
Glucose	96	70-99 mg/dL
Sodium	139	136-145 mEq/L
Potassium	4.8	3.5-5.0 mEq/L
Chloride	105	95-105 mEq/L
Total calcium	9.4	8.8-10.1 mg/dL
Amylase	44	44-132 U/L
Creatine phosphokinase	68	41-153 U/L
CRP	0.02	0.00-0.14 mg/dL
Tumor marker	Value	Reference range
CEA	1.3	0.0-5.0 ng/mL
CA 15-3	8.9	0.0-31.3 U/mL

We first used ddEC (epirubicin 120 mg/body [85 mg/m^2^; 94% dose], cyclophosphamide 800 mg/body [560 mg/m^2^; 93% dose]) as neoadjuvant chemotherapy with PEG-G-CSF injection on day three. Concomitant medications were the same as in Case 1. These concomitant medications were used in all cycles.

**Figure 2:**
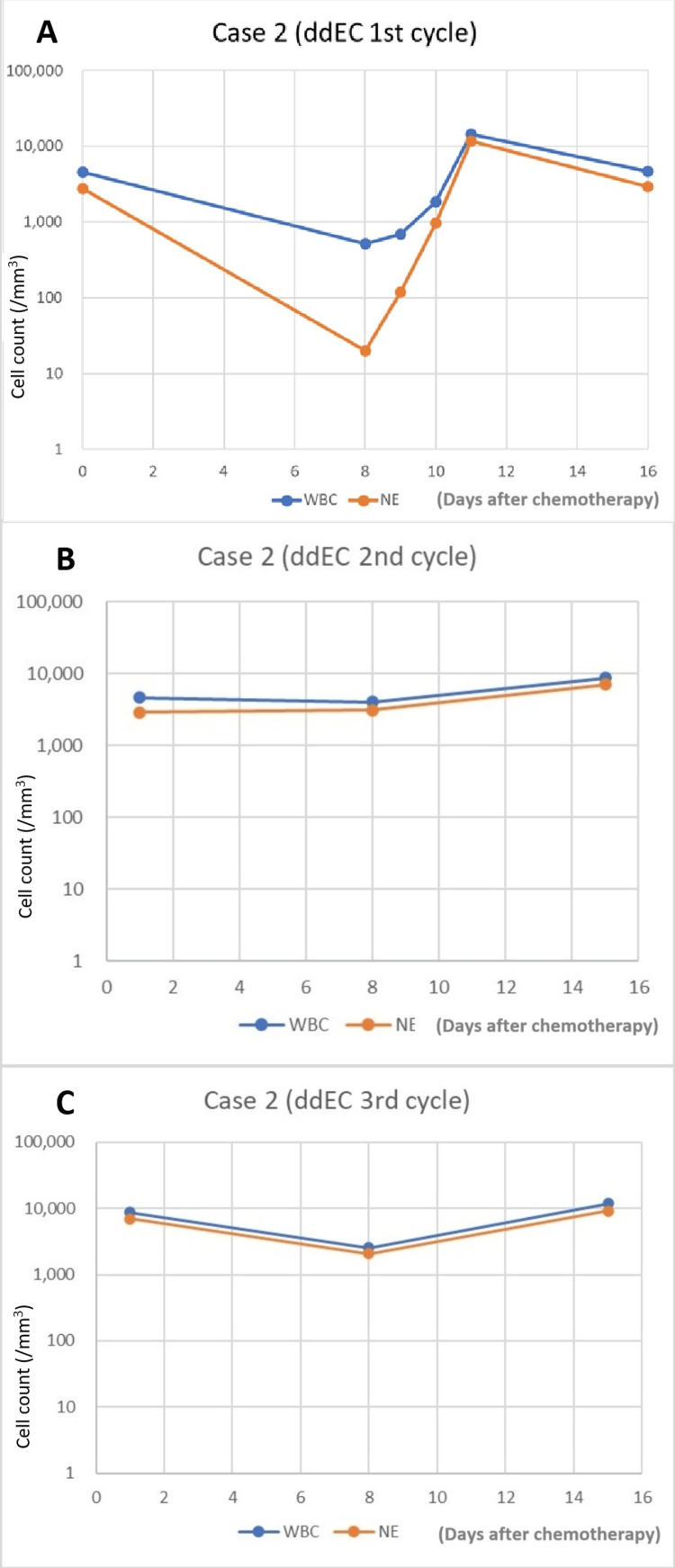
WBC and NE in Case 2 after PEG-G-CSF injection on day three (A) or day four (B and C) (A) ddEC, 1st cycle. (B) ddEC, 2nd cycle. (C) ddEC 3rd cycle ddEC: dose-dense epirubicin and cyclophosphamide; NE: neutrophil; PEG-G-CSF: pegylated granulocyte colony-stimulating factor; WBC: white blood cells

After the first ddEC administration, the patient showed mild fatigue and anorexia, but no medication was required. On day eight of the first cycle, routine blood testing was performed for the first ddEC administration on an outpatient basis. We observed extremely low WBC and NE, at 520/mm^3^ and 20/mm^3^, respectively (Figure 2A). Although no symptoms such as fever were observed, she was considered to be at high risk of FN and was admitted to the hospital urgently. The same day, G-CSF and the antibiotic MEPM (meropenem hydrate) were administered. A partition-type air purifier was also used. Blood tests the next day showed increased WBC and NE counts. MEPM was administered for two days, followed by LVFX. Due to leukocytosis and neutrophilia, G-CSF was discontinued after three consecutive days of administration, and the patient was discharged.

In the second cycle of ddEC therapy with a 16-18% reduced dose (epirubicin 100 mg/body [70 mg/m^2^; 78% dose], cyclopshosphamide 640 mg/body [450 mg/m^2^; 75% dose]) and PEG-G-CSF injection on day four, we observed increases in WBC and NE counts to 4,060/mm^3^ and 3,090/mm^3^, respectively (Figure 2B). Mild fatigue and anorexia appeared during the first several days of the second cycle of ddEC, but resolved with oral antiemetics. The patient was able to tolerate the chemotherapy with oral anti-nausea medications alone.

In the third cycle of ddEC chemotherapy with a dose reduced 8% from the first cycle (epirubicin 110 mg/body [77 mg/m^2^; 86% dose], cyclopshosphamide 720 mg/body [510 mg/m^2^; 85% dose]) and PEG-G-CSF injection on day four, WBC and NE counts were 2,560/mm^3^ and 2,060/mm^3^, respectively, on day eight (Figure 2C). The third cycle followed a similar clinical course to the second. No fever was observed during any of the three cycles.

Monocyte counts measured on day eight of the second and third cycles, especially in the second cycle, were higher than those measured on day seven of the first cycle (Table 4). With regular outpatient visits and examinations, since completing neoadjuvant chemotherapy, she has been alive for four years without a recurrence of breast cancer.

**Table 4:** Comparisons of monocytes in Case 2 at different times of PEG-G-CSF (day three of cycle [c]1 of ddEC therapy or day four of c2 and c3 of ddEC therapy) ddEC: dose-dense epirubicin and cyclophosphamide; PEG-G-CSF: pegylated granulocyte colony-stimulating factor

Comparisons of monocytes								
Day after CT (c1)	0	1	8	9	10	11	15	16
Monocytes (%)	3.7	NA	11.5	11.6	11.8	6.6	NA	6
Monocytes (/mm^3^)	168	NA	60	80	221	956	NA	280
Day after CT (c2)	0	1	8	9	10	11	15	16
Monocytes (%)	NA	6	10.8	NA	NA	NA	5.3	NA
Monocytes (/mm^3^)	NA	280	934	NA	NA	NA	458	NA
Day after CT (c3)	0	1	8	9	10	11	15	16
Monocytes (%)	NA	5.3	7	NA	NA	NA	6.8	NA
Monocytes (/mm^3^)	NA	458	189	NA	NA	NA	803	NA

Case 3: A 62-Year-Old Female

The patient with right-sided breast cancer had undergone a partial mastectomy and a sentinel lymph node biopsy. Pathological analysis showed invasive ductal carcinoma, defined as pT1 (invasive part, 16x10 mm) N0M0 stage I. Immunohistochemistry showed the following findings: ER- (J score 0); PgR- (J score 1); HER2- (1+); and Ki67 labeling index (71.7%). Height, weight, and body surface area before chemotherapy were as follows: 159.4 cm, 56.7 kg, and 1.58 m^2^ (Du Bois's formula), respectively. ECOG performance status was 0.

Table 5 shows the patient's laboratory data before breast surgery and adjuvant chemotherapy. These data indicated that the patient was relatively healthy and that preoperative chemotherapy would be unlikely to prove problematic.

**Table 5:** Laboratory data before breast surgery and chemotherapy - Case 3 ALT: alanine aminotransferase; APTT: activated partial thromboplastin time; AST: aspartate aminotransferase; CEA: carcinoembryonic antigen; CRP: C-reactive protein; PT: prothrombin time; PT-INR: prothrombin time-international normalized ratio; RBC: red blood cells; WBC: white blood cells; CA 15-3: cancer antigen 15-3

Lab data		
Complete blood count	Value	Reference range
WBC	5000	3300-8600/mm^3^
RBC	4.22 x 10^6^	3.86-4.92 x 10^6^/mm^3^
Hemoglobin	12.9	11.6-14.8 g/dL
Platelets	19.4 x 10^4^	15.8-34.8 x 10^4^/mm^3^
Differential WBC (count)	Value	Reference range
Neutrophils	2990	NA
Lymphocytes	1680	NA
Monocytes	150	NA
Eosinophils	130	NA
Basophils	50	NA
Differential WBC (%)	Value	Reference range
Neutrophils	59.8	37-72 (%)
Lymphocytes	33.6	20-50 (%)
Monocytes	3	4.1-10.6 (%)
Eosinophils	2.6	0.6-8.3 (%)
Basophils	1	0-1.3 (%)
Coagulation	Value	Reference range
PT	11.7	10.5-12.5 sec
PT-INR	0.99	NA
APTT	27.3	25-35 sec
Urinalysis	Value	Reference range
WBC	(1+)	(-)
Nitrite	(-)	(-)
Urobilinogen	(+/-)	(+/-)
Protein	(-)	(-)
pH	5.5	NA
Blood	(-)	(-)
Specific gravity	1.005	NA
Ketones	(-)	(-)
Bilirubin	(-)	(-)
Glucose	(-)	(-)
Blood chemistry	Value	Reference range
Total protein	7.2	6.6-8.1 g/dL
Albumin	4.7	4.1-5.1 g/dL
Total bilirubin	0.8	0.4-1.5 mg/dL
ALT	20	13-30 U/L
AST	11	7-23 U/L
Gamma-glutamyl transferase	36	9-32 U/L
Creatinine	0.56	0.8-1.3 mg/dL
Blood urea nitrogen	15.1	8-20 mg/dL
Uric acid	5	2.6-5.5 mgl/dL
Glucose	89	73-109 mg/dL
Sodium	140	138-145 mEq/L
Potassium	4	3.5-5.0 mEq/L
Chloride	106	101-108 mEq/L
Total calcium	9.1	8.8-10.1 mg/dL
Amylase	63	44-132 U/L
Creatine phosphokinase	127	41-153 U/L
CRP	0.08	0.00-0.14 mg/dL
Tumor marker	Value	Reference range
CEA	2.6	0.0-5.0 ng/mL
CA 15-3	21	0.0-31.3 U/mL

We used ddEC as adjuvant chemotherapy. The concomitant medications were the same as in Case 1. These concomitant medications were used in all cycles.

**Figure 3:**
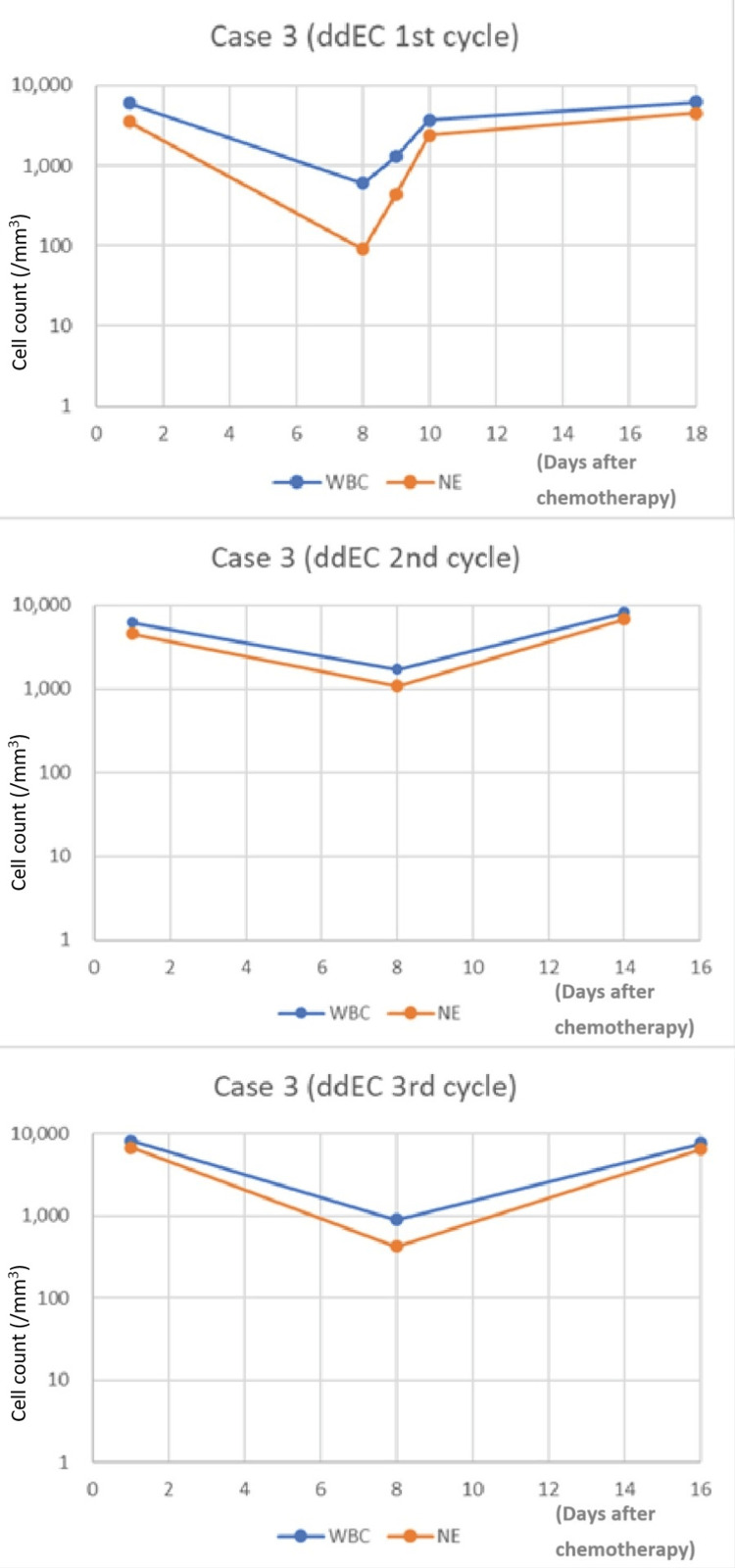
WBC and NE in Case 3 after PEG-G-CSF injection on day three (A) or day four (B and C) (A) ddEC, 1st cycle. (B) ddEC, 2nd cycle. (C) ddEC, 3rd cycle ddEC: dose-dense epirubicin and cyclophosphamide; NE: neutrophil; PEG-G-CSF: pegylated granulocyte colony-stimulating factor; WBC: white blood cells

In the first cycle of ddEC (epirubicin 140 mg/body [89 mg/m^2^; 98% dose], cyclopshosphamide 950 mg/body [560 mg/m^2^; 100% dose]) with PEG-G-CSF injection on day three, routine blood testing revealed decreases in WBC and NE counts on day eight, at 600/mm^3^ and 90/mm^3^, respectively (Figure 3A). Only mild nausea was observed after the first chemotherapy. Although no symptoms such as fever were seen, the decreases in WBC and NE counts suggested a high risk of FN. G-CSF was therefore administered. As antibiotics, oral LVFX was administered at the same time. Thrombocytopenia was also observed, and the platelet count was 58,000 (Grade 3). G-CSF was discontinued after three consecutive days of administration due to leukocytosis and neutrophilia.

In the second cycle of ddEC chemotherapy with around 90% of the first ddEC (epirubicin 130 mg/body [82 mg/m^2^; 91% dose], cyclopshosphamide 850 mg/body [538 mg/m^2^; 90% dose]) and PEG-G-CSF injection on day four, we observed increases in WBC and NE counts to 1,700/mm^3^ and 1,070/mm^3^, respectively (Figure 3B). Mild fatigue and constipation were observed during the second cycle.

In the third cycle of ddEC chemotherapy with the dose at about 100% dose of the first cycle (epirubicin 140mg/body [89 mg/m^2^; 98% dose], cyclopshosphamide 950 mg/body [560 mg/m^2^; 100% dose]) and PEG-G-CSF injection on day four, we observed increased WBC and NE counts, at 900/mm^3^ and 420/mm^3^, respectively, on day eight (Figure 3C). Mild fatigue and constipation were observed during the third cycle. No fever was observed during any of the three cycles.

Monocyte counts measured on day eight of the second and third cycles were higher than those on day 7 of the first cycle (Table 6). With regular outpatient visits and examinations, the patient has remained alive for four years since surgery without any recurrence of breast cancer.

**Table 6:** Comparisons of monocytes in Case 3 at different times of PEG-G-CSF injection (day three of cycle [c]1 of ddEC therapy or day four of c2 and c3 of ddEC therapy) ddEC: dose-dense epirubicin and cyclophosphamide; PEG-G-CSF: pegylated granulocyte colony-stimulating factor

Comparisons of monocytes							
Day after CT (c1)	1	8	9	10	14	16	18
Monocytes (%)	4.4	3.3	5.6	6.2	NA	NA	6.8
Monocytes (/mm^3^)	264	20	73	229	NA	NA	422
Day after CT (c2)	1	8	9	10	14	16	18
Monocytes (%)	6.8	3.6	NA	NA	5.8	NA	NA
Monocytes (/mm^3^)	422	61	NA	NA	470	NA	NA
Day after CT (c3)	1	8	9	10	14	16	18
Monocytes (%)	5.8	6	NA	NA	NA	6.6	NA
Monocytes (/mm^3^)	470	54	NA	NA	NA	502	NA

Case 4: A 72-Year-Old Female

This patient with right-sided breast cancer had undergone a reserved total mastectomy and sentinel lymph node biopsy. Pathological analysis showed invasive lobular carcinoma, pT2 (invasive part, 34 mm) N0M0 stage IIA. Immunohistochemistry showed the following findings: ER+ (Allred score: TS 8); PgR- (TS 0); HER2- (2+) (fluorescein in situ hybridization, FISH 1.0); Ki67 labeling index, 36.1%; and no E-cadherin expression. Height, weight, and body surface area before chemotherapy were as follows: 153 cm, 46.7 kg, and 1.41 m^2^ (Du Bois formula), respectively. ECOG performance status was 0.

Table 7 shows the laboratory data for this patient before breast surgery and chemotherapy. These data indicated that the patient was relatively healthy and that preoperative chemotherapy would not likely pose a problem.

**Table 7:** Laboratory data on admission - Case 4 ALT: alanine aminotransferase; APTT: activated partial thromboplastin time; AST: aspartate aminotransferase; CEA: carcinoembryonic antigen; CRP: C-reactive protein; PT: prothrombin time; PT-INR: prothrombin time-international normalized ratio; RBC: red blood cells; WBC: white blood cells; CA 15-3: cancer antigen 15-3

Lab data		
Complete blood count	Value	Reference range
WBC	8000	3300-8600/mm^3^
RBC	4.35 x 10^6^	3.86-4.92 x 10^6^/mm^3^
Hemoglobin	13.5	11.6-14.8 g/dl
Platelets	35.9 x 10^4^	15.8-34.8 x 10^4^/mm^3^
Differential WBC (count)	Value	Reference range
Neutrophils	5260	NA
Lymphocytes	1910	NA
Monocytes	350	NA
Eosinophils	430	NA
Basophils	50	NA
Differential WBC (%)	Value	Reference range
Neutrophils	65.7	37-72 (%)
Lymphocytes	23.9	20-50 (%)
Monocytes	4.4	4.1-10.6 (%)
Eosinophils	5.4	0.6-8.3 (%)
Basophils	0.6	0-1.3 (%)
Coagulation	Value	Reference range
PT	11.4	10.5-12.5 sec
PT-INR	0.95	NA
APTT	25.8	25-35 sec
Urinalysis	Value	Reference range
WBC	(-)	(-)
Nitrite	(-)	(-)
Urobilinogen	(+/-)	(+/-)
Protein	(-)	(-)
pH	5.5	NA
Blood	(+/-)	(-)
Specific gravity	1.012	NA
Ketones	(-)	(-)
Bilirubin	(-)	(-)
Glucose	(-)	(-)
Blood chemistry	Value	Reference range
Total protein	7.54	6.6-8.1 g/dL
Albumin	4.17	4.1-5.1 g/dL
Total bilirubin	1.1	0.4-1.5 mg/dL
ALT	20	13-30 U/L
AST	24	7-23 U/L
Gamma-glutamyl transferase	57	9-32 U/L
Creatinine	0.68	0.8-1.3 mg/dL
Blood urea nitrogen	15.5	8–20 mg/dL
Uric acid	7.4	2.6-5.5 mgl/dL
Glucose	103	73-109 mg/dL
Sodium	138	138-145 mEq/L
Potassium	4.6	3.5-5.0 mEq/L
Chloride	103	101-108 mEq/L
Total calcium	9.7	8.8-10.1 mg/dL
Amylase	67	44-132 U/L
Creatine phosphokinase	127	41-153 U/L
CRP	0.13	0.00-0.14 mg/dL
Tumor marker	Value	Reference range
CEA	2.6	0.0-5.0 ng/mL
CA 15-3	10.4	0.0-31.3 U/mL

We used basic EC as adjuvant chemotherapy in this case. The concomitant medications were the same as in Case 1. These concomitant medications were used in all cycles.

**Figure 4:**
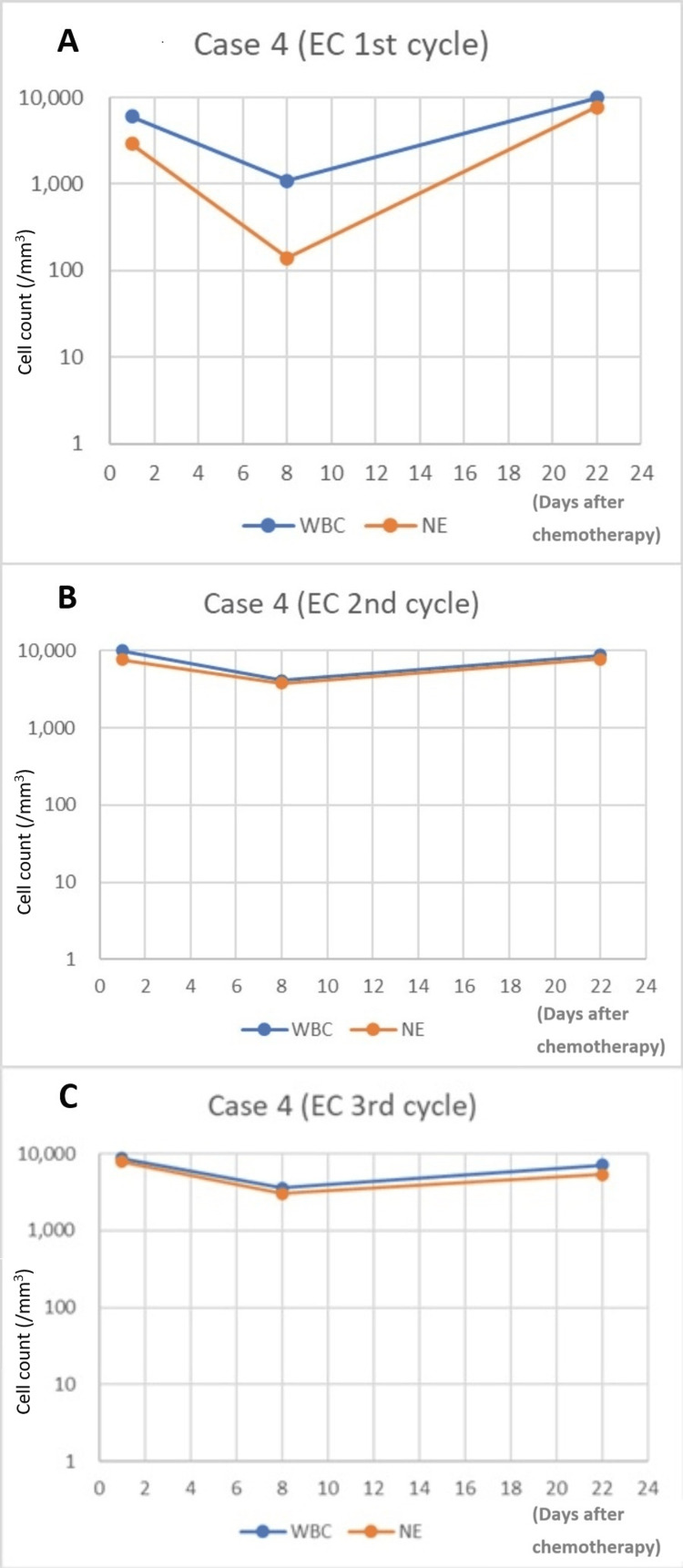
WBC and NE in Case 4 after PEG-G-CSF injection on day three (A) or day four (B and C) (A) EC, 1st cycle. (B) EC, 2nd cycle. (C) EC, 3rd cycle EC: epirubicin and cyclophosphamide; NE: neutrophil; PEG-G-CSF: pegylated granulocyte colony-stimulating factor; WBC: white blood cells

After the first EC therapy (epirubicin 120 mg/body [85 mg/m^2^; 95% dose], cyclophosphamide 800 mg/body [567 mg/m^2^; 95% dose]) with PEG-G-CSF injection on day three, the patient only displayed mild constipation. Routine blood sampling was performed on day eight of the first EC therapy on an outpatient basis. On day eight, we observed markedly low WBC and NE counts, at 1100/mm^3^ and 140/mm^3^, respectively (Figure 4A). Although no symptoms such as fever were observed, this was determined to be a risk factor for FN, and oral LVFX was administered.

On day 17, a sore throat and a slight fever of 37 °C were observed. On day 19, she was examined, and pharyngitis was suspected (body temperature: 37.6°C, WBC count: 13,300/mm^3^, NE count: 7,900/mm^3^, C-reactive protein [CRP]: 7.48 mg/dL, not listed in Figure 4A because there was determined to be no direct relationship with FN). Her general condition was good, and symptoms improved with the administration of non-steroidal anti-inflammatory drugs (NSAIDs).

Pharyngitis had improved by the time of the second cycle, and hence EC therapy was performed as scheduled (epirubicin 110 mg/body [78 mg/m^2^; 87% dose], cyclophosphamide 720 mg/body [511 mg/m^2^; 85% dose]). In the second cycle of EC chemotherapy with PEG-G-CSF injection on day four, we observed increases in WBC and NE counts, to 4100/mm^3^ and 3770/mm^3^, respectively (Figure 4B). The patient experienced mild fatigue and constipation during the second cycle of chemotherapy.

In the third cycle of EC chemotherapy (epirubicin 120mg/body [85 mg/m^2^; 95% dose], cyclophosphamide 800 mg/body [567 mg/m^2^; 95% dose]) with PEG-G-CSF injection on day four, WBC and NE counts were seen to increase to 4,000/mm^3^ and 3,020/mm^3^, respectively (Figure 4C). The patient also experienced mild fatigue and mild constipation during the third cycle. From the first to the third cycle of chemotherapy, only a slight fever due to pharyngitis was identified.

Monocyte counts measured on day eight of the second and third cycles were slightly higher than those measured on day seven of the first cycle (Table 8). With regular outpatient visits and examinations, the patient has remained alive for four years since surgery without any recurrence of breast cancer.

**Table 8:** Comparisons of monocytes in Case 4 at different times of PEG-G-CSF injection (day three of cycle [c]1 of EC therapy or day four of c2 and c3 of EC therapy) EC: epirubicin and cyclophosphamide; PEG-G-CSF: pegylated granulocyte colony-stimulating factor

Comparisons of monocytes			
Day after CT (c1)	1	8	22
Monocytes (%)	4.9	6	7.3
Monocytes (/mm^3^)	294	66	730
Day after CT (c2)	1	8	22
Monocytes (%)	7.3	2	5.4
Monocytes (/mm^3^)	730	82	437
Day after CT (c3)	1	8	22
Monocytes (%)	5.4	4	9.8
Monocytes (/mm^3^)	437	144	696

In all four cases, WBC and NE counts on day seven or eight (second and third cycle of Case 1 on day seven) following PEG-G-CSF injection on day four to prevent FN after breast cancer chemotherapy were much greater than those after PEG-G-CSF injection on day three. Notably, chemotherapy doses were the same in cycles one and three, allowing direct comparison of WBC and NE counts between both cycles. However, it should be noted that in Case 2, the chemotherapy dose in the third cycle was approximately 8% lower than that in the first cycle (Table 9).

**Table 9:** Summary of the chemotherapies and clinical courses of the four cases ddEC: dose-dense epirubicin and cyclophosphamide; EC: epirubicin and cyclophosphamide; FEC100: 5-fluorouracil 500 mg/m^2^, epirubicin 100 mg/m^2^, and cyclophosphamide 500 mg/m^2^; PEG-G-CSF: pegylated granulocyte colony-stimulating factor

Case	Age, years	Regimen	Cycle	Dose of chemotherapy	PEG-G-CSF injection	NE count on day 7 or 8 (/mm^3^)	WBC count on day 7 or 8 (/mm^3^)	Usage of antibiotics	Usage of G-CSF
1	55	FEC100	1	94%	Day 3	190	1,050	Yes	No
			2	86%	Day 4	11,800	13,000	No	No
			3	94%	Day 4	13,100	14,200	No	No
2	41	ddEC	1	93-94%	Day 3	20	520	Yes	Yes
			2	75-78%	Day 4	3,090	4,060	No	No
			3	85-86%	Day 4	2,060	2,560	No	No
3	62	ddEC	1	98-100%	Day 3	90	600	Yes	Yes
			2	90-91%	Day 4	1,070	1,700	No	No
			3	98-100%	Day 4	420	900	No	No
4	72	EC	1	95%	Day 3	140	1,100	Yes	No
			2	85-87%	Day 4	3,770	4,100	No	No
			3	95%	Day 4	3,020	3,600	No	No

## Discussion

PEG-G-CSF is widely used as prophylaxis for FN after breast cancer chemotherapy, allowing increased dose intensity. The effect of this prophylaxis in elevating the NE is very high and long-lasting. PEG-G-CSF is recommended for use in regimens with a >20% risk of FN according to the clinical practice guidelines of the Japanese Society of Clinical Oncology [4]. However, FN still occurs in 2.8-16% of cases even with such prophylactic use of PEG-G-CSF [1,2]. In fact, all four cases in this report showed severe neutropenia, at 20-190/mm^3^ on day seven or eight after chemotherapy. After chemotherapy, monocyte counts increase more rapidly than neutrophil counts, and hence an increase in monocytes is considered to offer a predictive marker of an increase in neutrophils [6]. Except for Case 1, monocyte counts on days seven or eight of the first cycle were increased compared with those on days seven or eight of the second and third cycles. In Case 1, the neutrophil count was already elevated on day seven of the second and third cycles, so the peak day of monocytosis may have occurred before day seven.

Several reports have examined more effective use of this prophylactic agent. Those reports studied the timing of PEG-G-CSF injection after chemotherapy for lymphoma and breast cancer. Regarding lymphoma, two reports have been published. In the first, the results of PEG-G-CSF injection on day two and day four were compared in R-CHOP-14 therapy for lymphoma [7]. Day four injection appeared more effective for reducing severe leukocytopenia and death during leukocytopenia. In the second study, PEG-G-CSF injection on days zero, two, or four, and G-CSF injection for seven or 10 consecutive days were compared for solid tumor and non-Hodgkin’s lymphoma treated using intermediate and high-risk therapies [1]. PEG-G-CSF injection on day four again appeared more effective for reducing leukocytopenia, infection, and days in hospital.

Two studies have been published regarding breast cancer [2,8]. In the first, 87 Japanese breast cancer patients receiving intermediate-risk breast cancer chemotherapy (including EC; n = 78) were administered 318 cycles of PEG-G-CSF. Of these, 14 (16%) developed FN. The results showed that PEG-G-CSF administered on day two was more closely associated with FN than administration on day three or later (odds ratio: 11.0, p = 0.009) [2]. In the second study, the multicenter prospective GAIN trial randomly assigned 351 patients with lymph node-positive breast cancer receiving chemotherapy with epirubicin, paclitaxel, and cyclophosphamide to receive PEG-G-CSF on day two (P2; n = 174) or day four (P4; n = 177). This study failed to confirm any clear benefit from PEG-G-CSF on day four compared to day two [8].

The reasons for the discrepancies in the apparent optimal timing of PEG-G-CSF injection in these reports remain unclear, but could be due to pharmacokinetics. For anticancer drugs, individual differences in adverse events can also arise due to differences in patient genetics. The risk of FN associated with breast cancer chemotherapy may be attributed to individual differences in the metabolism of anticancer drugs, which may be the cause of the discrepancies seen in previous studies. If individual differences exist in the ability to metabolize anticancer drugs to an inactive form and the proportion of individuals with slower metabolization included in the study population remains unknown, the results of administrations on days two and three may not match. To eliminate such individual variability as much as possible, we decided to try a different method to determine the optimal timing of PEG-G-CSF administration.

Previous studies have compared administration on days two and three with later days or day two and day four between groups. These groups may contain individuals with different clinical and genetic backgrounds. Given the frequency of FN, we believe that chemotherapy-sensitive individuals are relatively few in number compared to their chemotherapy-resistant counterparts. We therefore believe that determining the appropriate timing of administration or PEG-G-CSF by conducting comparisons between groups may be difficult. However, the present study compared results from the same patients receiving PEG-G-CSF at different times. We found that in this small cohort, PEG-G-CSF administered on day three after initial chemotherapy showed markedly lower WBC and NE counts on day eight. These cases were considered at high risk of FN.

In this report, administration of PEG-G-CSF was changed to day four in the next cycle for each of the four patients. As a result, administration on day four resulted in significantly higher WBC and NE counts. Hence, we believe that administration on day four was more effective for preventing FN in these cases. How WBC and NE change following a change in the day of PEG-G-CSF administration remains unclear. Anticancer drug in WBCs and neutrophils when PEG-G-CSF was administered on day four was thought to be metabolized to a greater extent than with administration on day three, and hence the WBCs and NEs that proliferated in response to PEG-G-CSF did not undergo cell death and instead effectively increased in number.

As for other drugs, one example of how the metabolism of an anticancer drug differs due to genetic polymorphisms is irinotecan, an anticancer drug used in various cancers such as colorectal cancer and breast cancer. The active metabolite of irinotecan is SN-38, which is detoxified by an enzyme called UDP-glucuronosyltransferase, encoded by *UGT1A1*. Some variants of *UGT1A1* are known to delay the excretion of SN-38 [9-11]. In addition, 5FU-based anticancer drugs are used against many solid tumors, and 5FU is known to be degraded by the enzyme encoded by the detoxification metabolic gene *DPD*. Recent studies have clarified four types of polymorphisms in *DPD*, reportedly offering markers for predicting the risk of side effects from 5-FU-based anticancer drugs [12,13].

The genes *UGT2B7* and *AKR1B10* are known to be involved in epirubicin metabolism [14, 15]. Cyclophosphamide is metabolized by cytochrome P450 in the liver and other organs to an activated form, and the glutathione gene *GSTP1* is involved in the decomposition of cyclophosphamide. Variants in the genes that code for these enzymes seem likely to exist [16]. In the future, identification of variants in these genes and confirmation of correlations with these phenomena may enable the prediction of the risk of FN.

## Conclusions

Pegfilgrastim (PEG-G-CSF) is commonly used as prophylaxis for FN in high-risk chemotherapy regimens for breast cancer. However, the optimal timing of PEG-G-CSF injections after chemotherapy has not been established. Our findings showed that when the administration timing of PEG-G-CSF was changed from day three to day four, NE count increased on day seven or eight in all four cases. Furthermore, there have been no reports investigating the administration timing of PEG-G-CSF using this analytical method. However, due to the small number of cases analyzed in this series and the confounding factor of the chemotherapy dose in the third cycle being approximately 8% less than that in the first cycle in Case 2, it is difficult to generalize the finding that administering PEG-G-CSF on the fourth day is more effective than administering it on the third day in preventing FN during breast cancer chemotherapy. We believe that further accumulation of cases is required to validate our findings.

## References

[1] Weycker D, Li X, Figueredo J, Barron R, Tzivelekis S, Hagiwara M (2016). Risk of chemotherapy-induced febrile neutropenia in cancer patients receiving pegfilgrastim prophylaxis: does timing of administration matter?. Support Care Cancer.

[2] Hayama T, Sakurai K, Miura K (2018). Optimal timing for pegfilgrastim administration in Japanese breast cancer patients receiving intermediate-risk chemotherapies. Int J Clin Pharm.

[3] Kanbayashi Y, Ishikawa T, Kanazawa M (2018). Predictive factors in patients eligible for pegfilgrastim prophylaxis focusing on RDI using ordered logistic regression analysis. Med Oncol.

[4] Japanese Society of Clinical Oncology (2022). The Guidelines for Appropriate Use of G-CSF, Second Edition.

[5] Hansson EK, Friberg LE (2012). The shape of the myelosuppression time profile is related to the probability of developing neutropenic fever in patients with docetaxel-induced grade IV neutropenia. Cancer Chemother Pharmacol.

[6] Hansson M, Svensson A, Engervall P, Björkholm M, Gruber A, Söderström T (1995). Increase of monocytes predicts mobilization of peripheral stem and progenitor cells after chemotherapy followed by G-CSF administration. Eur J Haematol.

[7] Zwick C, Hartmann F, Zeynalova S (2011). Randomized comparison of pegfilgrastim day 4 versus day 2 for the prevention of chemotherapy-induced leukocytopenia. Ann Oncol.

[8] Loibl S, Mueller V, von Minckwitz G (2011). Comparison of pegfilgrastim on day 2 vs. day 4 as primary prophylaxis of intense dose-dense chemotherapy in patients with node-positive primary breast cancer within the prospective, multi-center GAIN study: (GBG 33). Support Care Cancer.

[9] Ando Y, Saka H, Ando M (2000). Polymorphisms of UDP-glucuronosyltransferase gene and irinotecan toxicity: a pharmacogenetic analysis. Cancer Res.

[10] Innocenti F, Undevia SD, Iyer L (2004). Genetic variants in the UDP-glucuronosyltransferase 1A1 gene predict the risk of severe neutropenia of irinotecan. J Clin Oncol.

[11] Minami H, Sai K, Saeki M (2007). Irinotecan pharmacokinetics/pharmacodynamics and UGT1A genetic polymorphisms in Japanese: roles of UGT1A1*6 and *28. Pharmacogenet Genomics.

[12] Henricks LM, Lunenburg CA, Meulendijks D (2015). Translating DPYD genotype into DPD phenotype: using the DPYD gene activity score. Pharmacogenomics.

[13] Meulendijks D, Henricks LM, Sonke GS (2015). Clinical relevance of DPYD variants c.1679T>G, c.1236G>A/HapB3, and c.1601G> as predictors of severe fluoropyrimidine-related toxicity. Clinical relevance of A: systematic review and meta-analysis of individual patient data. Lancet Oncol.

[14] Innocenti F, Iyer L, Ramírez J, Green MD, Ratain MJ (2001). Epirubicin glucuronidation is catalyzed by human UDP-glucuronosyltransferase 2B7. Drug Metab Dispos.

[15] Zhong L, Shen H, Huang C, Jing H, Cao D (2011). AKR1B10 induces cell resistance to daunorubicin and idarubicin by reducing C13 ketonic group. Toxicol Appl Pharmacol.

[16] Sugishita M, Imai T, Kikumori T (2016). Pharmacogenetic association between GSTP1 genetic polymorphism and febrile neutropenia in Japanese patients with early breast cancer. Breast Cancer.

